# Tissue-specific regulation of CXCL9/10/11 chemokines in keratinocytes: Implications for oral inflammatory disease

**DOI:** 10.1371/journal.pone.0172821

**Published:** 2017-03-02

**Authors:** Alison Marshall, Antonio Celentano, Nicola Cirillo, Michael McCullough, Stephen Porter

**Affiliations:** 1 University College London, UCL Eastman Dental Institute, London, United Kingdom; 2 Department of Neurosciences, Reproductive and Odontostomatological Sciences, University Federico II of Naples, Naples, Italy; 3 Melbourne Dental School and Oral Health CRC, The University of Melbourne, Victoria, Australia; Katholieke Universiteit Leuven Rega Institute for Medical Research, BELGIUM

## Abstract

The IFN-γ-inducible chemokines CXCL9, CXCL10, and CXCL11 play a key role in many inflammatory conditions, particularly those mediated by T cells. Therefore, the production of these chemokines in peripheral tissues could be instrumental in the pathophysiology of tissue-specific immunological diseases such as oral lichen planus (OLP). In the present study, we assessed the production of keratinocyte-derived CXCL9/10/11 under basal and inflammatory conditions and investigated whether these chemokines were involved in the pathogenesis of OLP. We used semi-quantitative PCR, ELISA, chemotaxis assays, and fluorescence-activated cell sorting (FACS) to assess the expression and functional role of CXCL9/10/11 in oral keratinocytes (three strains of normal human oral keratinocytes (NHOK), and the H357 oral cancer cell line) in the presence or absence of IFN-γ. CXCL9/10/11 were also assessed in tissues from normal patients and those with oral lichen planus (OLP). The time course study in oral keratinocytes treated with IFN-γ showed that expression of CXCL9/10/11 chemokines was significantly enhanced by IFN-γ in a time-dependent manner. In particular, CXCL10, a prominent chemokine that was overexpressed by IFN-γ-stimulated NHOK, was able to effectively recruit CD4 lymphocytes, mainly CD4+CD45RA- cells. Significantly higher levels of CXCL9/10/11 were found in tissues from patients with OLP compared to normal oral mucosa. Taken together, the results demonstrate that normal oral keratinocytes produce chemotactic molecules that mediate T cell recruitment. This study furthers understanding of chemokine production in oral keratinocytes and their role in the pathophysiology of oral mucosa, with particular relevance to OLP.

## Introduction

Interferon-γ (IFN-γ), also known as immune type II interferon, is a pleiotropic cytokine secreted by CD4 Th1, CD8, γδ T, and natural killer (NK) cells. Its main functions encompass regulation of the immune system and the control of infectious disease. This Th1 cytokine plays an essential role in both the innate and adaptive phases of an immune response [1,2]. Interestingly, IFN-γ and other Th 1 cytokines have been demonstrated to regulate the immunological activity in T-cell-mediated inflammation of the oral mucosa, such as in OLP [3,4]. One of the mechanisms by which IFN-γ exerts its immunological function is by inducing the production of a subset of pro-inflammatory chemokines that stimulate leukocyte migration and takes part in the regulation of leukocyte trafficking through lymphoid tissues [5,6].

Three such chemokines induced by IFN-γ, i.e. monokine induced by IFN-γ (MIG) (CXCL9), IFN-induced protein-10 (IP-10) (CXCL10) and IFN-γ induced T-cell attractant chemokine (I-TAC) (CXCL11), belong to the CXC family and are characterised by the lack of a Glu-leu-arg (ELR) motif [7]. CXCL9/10/11 all bind the CXCR3 receptor [8–10], which is predominately expressed on activated/memory CD4/CD8 cells [8,11,12] that are associated with a Th1 phenotype [13], and on dendritic cells and natural killer cells, but also fibroblasts and smooth muscle, epithelial and endothelial cells [14]. These IFN-γ-inducible chemokines can be produced by a number of different cell types including haemopoetic cell types, e.g. macrophages and neutrophils [15,16] and non-haemopoetic cell types, such as endothelial cells [17], fibroblasts [15,18], and epithelial cells [19–21], including skin keratinocytes [9,22,23], some human oral SCC cell lines [24], as well as one immortalized oral keratinocyte cell line [18]. However, the expression of CXCR3-binding chemokines in primary oral keratinocytes and normal oral mucosal tissues has not been convincingly demonstrated so far.

The production of CXCL9, CXCL10 and CXCL11 is associated with many T cell mediated conditions like organ rejection [15,25,26], autoimmune conditions [27], rheumatoid arthritis [12,28], inflammatory bowel diseases [12,29] and airway inflammation [30]. These chemokines are also characteristic of certain skin inflammatory disorders, such as contact hypersensitivity [31–34], interface dermatitis [35], Lichenoid graft-versus-host disease (liGVHD) [35] and lichen planus [9,18,31, 36–38], where the chemokines are produced in abundance in the diseased tissues. The identification of these chemokines in oral inflammation [39], suggests that these are also influential in the infiltration of T cells to oral mucosa. As OLP is a condition characterised by a large T cell infiltrate localised in a band-like pattern directly beneath the basal epithelium, and because IFN-γ is instrumental in the immunopathogenesis of OLP [40], we hypothesized that an IFN-γ-induced local production of pro-inflammatory chemokines by oral keratinocytes could potentially represent an important mechanism involved in T cell recruitment.

In the present study, we investigated the expression pattern of the CXCR3-binding chemokines CXCL9/10/11 in normal human oral keratinocytes (NHOK) after IFN-γ treatment, in order to assess whether these chemokines can be induced under IFN-γ stimulation in the oral epithelium, and thus may be able to promote T cell migration to the oral epithelial tissues. The potential role that these three chemokines play in oral inflammation was evaluated by assessing the relative level of CXCR3-binding chemokines mRNA in OLP and normal oral mucosal tissue.

## Materials & methods

### Patients

All OLP tissue was collected from patients that were attending the Oral Medicine Clinic, Eastman Dental Institute. The diagnosis of OLP was assessed clinically by two oral medicine specialists (M.M. and S.P.) according to well established criteria,and confirmed histopathologically. The medical history of the patients included in this study was carefully revised, and didn’t include any concomitant pharmacological treatment, hematological anomalies or systemic disease. All normal oral mucosa was obtained from patients attending the Oral Surgery Clinic, Eastman Dental Institute for routine third molar extraction. A total number of 54 samples (36 OLP and 18 NOM) were collected. The internal Ethical Committee of the UCL Eastman Dental Institute approved the study protocol, which was performed in accordance with the tenets of the Declaration of Helsinki. All patients provided written informed consent.

### Cell culture techniques

#### Normal human oral keratinocytes (NHOK) cell culture

Normal oral mucosal tissue was obtained for this study from healthy patients. Three different NHOK strains (NHOK1, NHOK2, NHOK3) were isolated from the excised normal tissue by separating the connective tissue. The samples were cut into approximately 1mm^3^ pieces and culturing at 37°C /5% CO_2_ in keratinocyte basal medium-2 containing the recommended growth supplements (Biowittaker, Wokingham, UK). The epithelial cells were then detached using 0.25% trypsin-1mM EDTA. The viability of the keratinocytes was confirmed by trypan blue exclusion.

#### H357 cell culture

The oral squamous cell carcinoma cell line, H357, was established by Prime et al [41], from a primary explant of a tongue squamous cell carcinoma. This cell line was grown in the same medium as described for the NHOK.

### IFN-γ cell treatment assay

In a modification of the method utilised by Altenburg *et al* [42], the NHOK1, NHOK2, NHOK3 and the H357 cell line (at 2^nd^ or 3^rd^ passage) were seeded at 8x10^4^cells/ well in a Falcon 6 well plate (Becton Dickinson, Oxford, UK) with 3mls of KBM-2 medium containing no hydrocortisone. The cells were incubated for at least 3–5 days until cell culture was 60–80% confluent. We set up the optimal experimental conditions in preliminary experiments with dose-response curves. Medium containing human recombinant 1000U/ml IFN-γ (catalogue number I3265, purity ≥ 98%, Sigma–Aldrich, Poole, UK) was added to 3 wells and control cell culture medium only was added to the remaining 3 wells. The 1000U/ml concentration of IFN-γ had been successfully used by previous studies to stimulate keratinocytes in vitro [42–45]. The cells were incubated for 48hrs or, in the case of the H357 time course, for the following time-points: 3hrs, 6hrs, 9hrs, 24hrs, 48hrs and 72hrs. The supernatant was extracted, centrifuged and stored at –70°C. The adherent cells were washed with PBS (Gibco Life Technologies, Paisley, UK) before 0.5ml of Trireagent were added. The suspension was then removed and stored at –70°C. The RNA was isolated as described below.

### mRNA isolation and semi-quantitative RT-PCR

OLP and normal oral mucosa (NOM) tissue were obtained and prepared for RNA isolation. RNA isolation and cDNA synthesis of NHOK, H357, NOM and OLP tissue was carried out. The RNA was extracted according to the manufacturer’s instructions, utilising 2ul Pellet Paint Co-precipitant (Novagen, Nottingham, UK) to visualise the RNA pellet. The purified RNA was dissolved into 25ul DEPC water (Ambion, Austin, US) and stored at -70°C.

All procedures for the cDNA Single strand synthesis were carried out on ice. 2ul of RNA was added to 4ul deoxynucleotides (dNTPs) (2.5mM) (Sigma), 2ul of random hexamers (50um) (Ambion, Texas, USA) and 9.5ul dH2O. This was incubated at 70°C for 3 minutes and allowed to cool at room temperature. Then 1ul of RNAaseIN (Ambion, Austin, Texas, USA), 2ul 10x MuLVRT buffer and 0.5ul M-MuLVRT (200U/ul) (Boehringer-Mannheim, Germany) was added and incubated at 42°C for 1 hour. cDNA samples were stored at –20°C.

CXCL9 (5’-ccaacaccccacagaagtgc–3’, 5’-gccagcacctgctctgagac-3’),

CXCL10 (5’- gccaattttgtccacgtgttg-3’, 5’-aaagaatttgggccccttgg-3’),

CXCL11 (5’-ggcttccccatgttcaaag –3’, 5’-cagatgcccttttccaggac-3’) and primers were generated for use in this study (Genosys-Sigma, Poole, UK). The thermocycler (Techne Genius, Cambridge, UK) parameters utilised were at 94°C for 45secs, 57°C for 45secs, 72°C for 45secs.

CXCL9/10/11 primers (as described above) were utilised with QuantamRNA 18S internal standards (Ambion, Texas, USA). The primers utilised for the study of housekeeping expression encoded a region of 18S ribosomal RNA (5’- tttcggaactgaggccatga-3’, 5’- gcatgccagagtctcgttcg -3’).

For each primer the linear range was determined by repeating the above reaction with optimised magnesium concentration for each primer and stopping the reaction at 15, 17, 19, 21, 23, 25, 27, 29, 31, 33 and 35 cycles. The mid-point of each linear range was determined by using intensity analysis of the bands with AlphaImager software, and this cycle length was utilised for each primer in subsequent reactions. 18S primer and 18S Competitor primers (Ambion, Texas, USA) were combined to ratios 1:9, 2:8 and 3:7 respectively. For each of the primers (CXCL9/10/11) 4μl of the 18S primer competitor mix was added to the RT-PCR reaction and the results were compared to the reactions containing the specific primers without any 18S primer. The reaction that had the same level of specific primer expression as that without 18S primer added was selected for quantification. The band intensity of the 18S and of the specific primer was quantified in each sample with Phoretix 1D software (Phoretix, Newcastle, UK).

### Enzyme-linked immunosorbant assay (ELISA)

A 96 well maxisorp-surface immunoplate (Nunc, Denmark) was coated overnight with a monoclonal antibody against the human protein to be studied. The plate was then washed 3 times with wash buffer (2.5mMNa_2_HPO_4_ (BDH), 0.5mM NaCl (BDH), 7.5mM NaH_2_PO_4_.2H_2_O (BDH) and 0.1% of Tween 20 (BDH). 100ml of cell supernatant or positive control (in a range of dilutions to obtain a standard curve) was added and incubated for 2 hours at room temperature then washed. A biotinylated antibody was used as a secondary antibody; 100μl of this antibody, diluted to an appropriate concentration, was added to each well. The plate was sealed and incubated for 1 hour at room temperature, then washed 3 times. Bound secondary antibody was detected by adding 100μl avidin-HRP (Dako, Denmark) [diluted 1:4000] and incubating for 30 minutes at room temperature. 25ml H_2_O_2_ was added to OPD (1 tablet of o-phenyl diaminazadine (Sigma, Poole, Dorset) in 25ml of 34.7mM citric acid, 66.7mM Na_2_HPO_4_) and 100μl of this solution was dispensed to each well immediately and incubated at room temperature for 15 mins. The reaction was stopped by adding 100ml of 1M sulphuric acid to the wells and the absorbance measured at 490nM. Chemokine concentration in the supernatant was then extrapolated from the standard curve generated from standards using Revelation software (Dynex Technologies, Virginia, US) attached to an ELISA plate reader (Dynex Technologies, Virginia, US).

### Chemotaxis assay

Peripheral blood mononuclear cells (PBMC) were prepared from fresh blood obtained from healthy patients. The lymphocyte separation was carried using Ficoll-Paque (Amersham) according to the manufacturers instructions. Peripheral blood lymphocytes obtained were incubated for 1hr in RPMI-1640 (Gibco Life Technologies, Paisley, UK) plus 5% foetal bovine serum (Sigma, Poole, UK), and those cells remaining in suspension were adjusted to a density of 5x10^6^ cells/ml.

The cells were then migrated towards 1μg/ml recombinant human CXCL10 or 100mM SDF-1alpha (R&D Systems, Minneapolis, US) in a transwell migration assay. 600μl of cell culture supernatant from each sample was added to the bottom chamber of Corning Co-star 5μm pore transwells (BDH, UK) in triplicate. 100μl of PBL was added to the top chamber. The transwells were then incubated for 3 hours at 37°C in atmosphere containing 5% CO_2_. After migration, the cells that passed through the membrane were collected and incubated with allophycocyanin conjugated anti-human CD45RA (Clone HI100) and Cy-chrome conjugated mouse anti-human CD4 (Clone RPA-T4) (Both BDPharmingen, San Diego, US) for 30 mins at 3μl /10^6^ cells. The migrated cell populations were then analysed using a florescence activated cell sorter (FACS) machine (Becton Dickinson, Oxford, UK).

### Statistical analysis

Unless otherwise specified all the experiments were performed at least in triplicate. All p values in studies included in this paper were obtained by executing paired Student’s T test upon the data, unless otherwise stated.

## Results

### Time course study of CXCL9/10/11 chemokine expression in keratinocytes treated with IFN-γ

**Fig 1:**
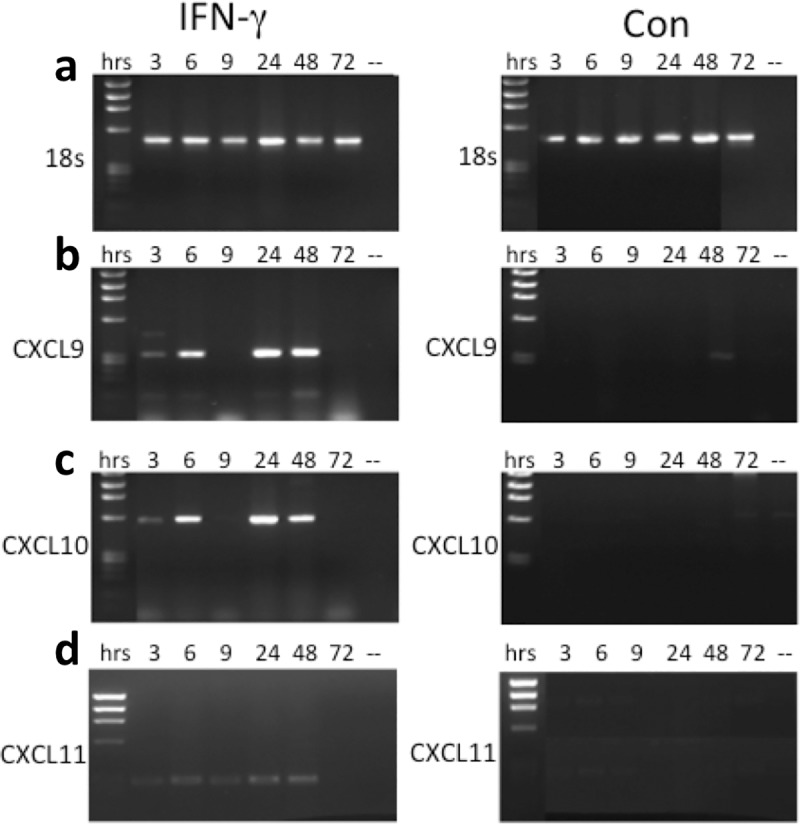
**18S (a), CXCL9 (b), CXCL10 (c) and CXCL11 (d) mRNA expression in the H357 cell line.** (ifn) = cells that were treated with IFN-γ for 3, 6, 9, 24, 48 or 72 hours; (con) = control cells that were left untreated over the same time period.

The production of CXCL9/10/11 chemokines in oral mucosal keratinocytes was first assessed over time in preliminary experiments using the keratinocyte cell line H357. After three hours of IFN-γ treatment, CXCL10 mRNA levels were detectable in H357 cells, with a peak at 24 hours. In contrast, the control cells showed virtually undetectable mRNA levels over the same time period. CXCL9 mRNA transcripts demonstrated a similar pattern to CXCL10, with a biphasic pattern showing a rapid induction of mRNA in the stimulated cells followed by a second peak at 24/48 hours (Fig 1). CXCL11 underwent a time-dependent increase over 48hrs, however mRNA transcripts were absent after 72hrs for all chemokines.

**Fig 2:**
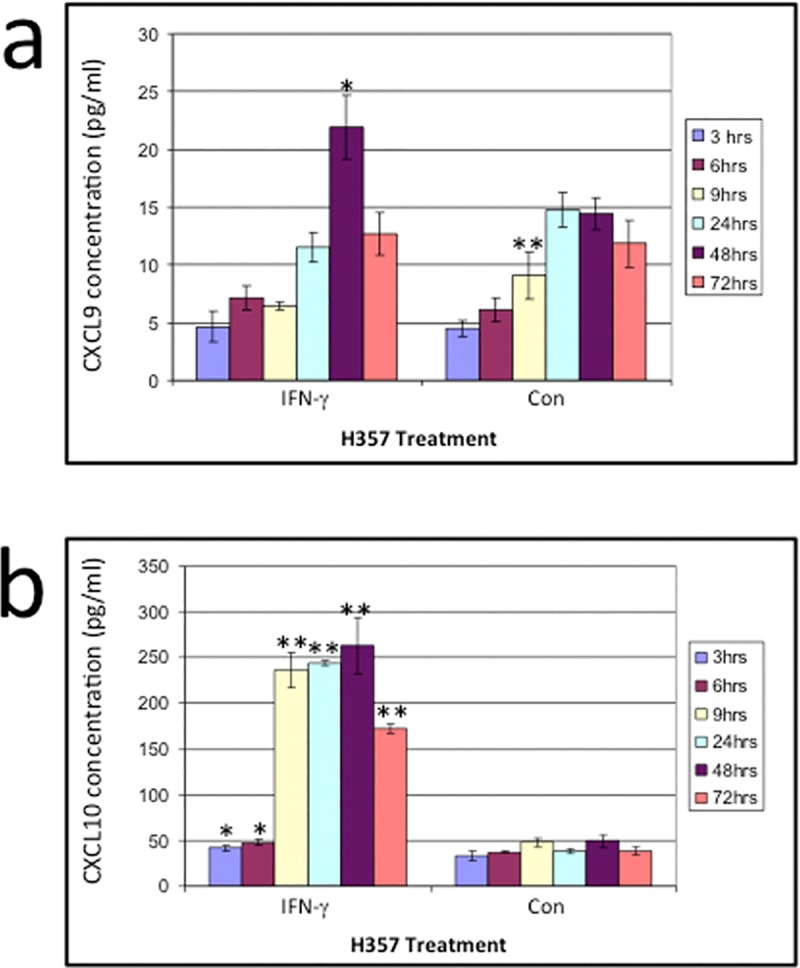
**a) The concentration of CXCL9 produced by the H357 cell line stimulated with IFN-γ for 3, 6, 9, 24, 48 and 72 hours. b) The concentration of CXCL10 produced by the H357 cell line without (con) or stimulated with IFN-γ (ifn) for 3, 6, 9, 24, 48 and 72 hours.** Significant differences in chemokines production between untreated cells and IFN-γ treated cell line are indicated as * = p<0.05 or ** = p<0.01. The ELISA is the result of triplicate experiments, shown with ±SD.

Interestingly, both CXCL9 and CXCL10 mRNA levels at 9 hours was virtually undetectable, and this prompted us to confirm if these chemokines were indeed expressed at the protein level. ELISA revealed a significant increase of CXCL10 protein at 9 hours, with a fairly stable expression of CXCL9 protein at the same time point. CXCL10 production reached a peak of 262.6pg/ml after 48 hours of IFN-γ stimulation, with a significant increase starting as early as 3 and 6 hours after stimulation (Fig 2B). The concentration of CXCL9 produced by H357 cells also reached a peak after 48 hours of IFN-γ incubation, but in contrast to CXCL10 this was the only time point where the concentration of CXCL9 was significantly higher than control cells (Fig 2A). The concentration of CXCL9 produced in IFN-γ-treated cells was lower than CXCL10 at all time points and appeared to undergo a slower increase.

Taken together, the data show that expression of CXCL9/10/11 in H357 cells can be significantly enhanced by IFN-γ in a time-dependent manner, with a peak after 48 hours.

### Induction of CXCL9/10/11 chemokines in NHOK

**Fig 3:**
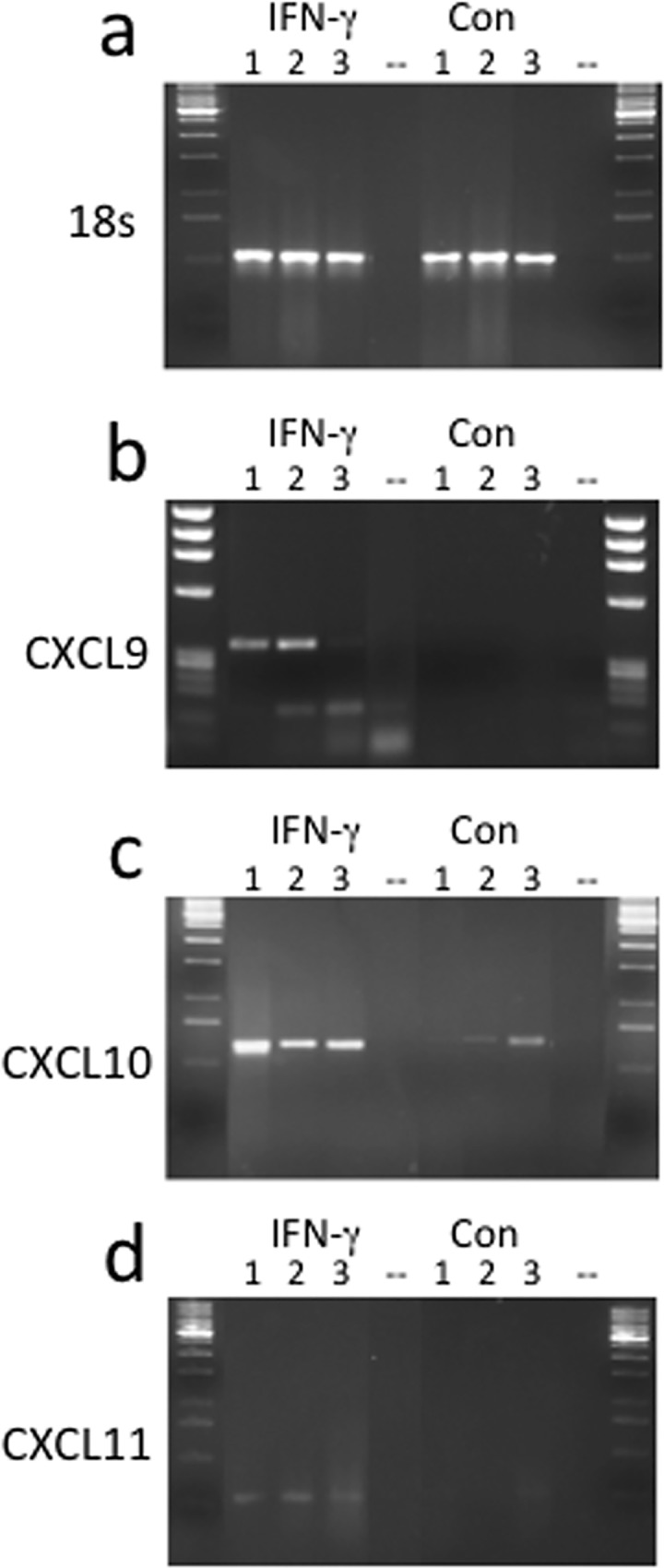
**18S (a), CXCL9 (b), CXCL10 (c) and CXCL11 (d) mRNA expression.** mRNA expression in 3 different normal human oral keratinocytes (NHOK), either IFN-γ treated for 48 hours (ifn) or control cells that were left untreated for 48hrs (con).

**Fig 4:**
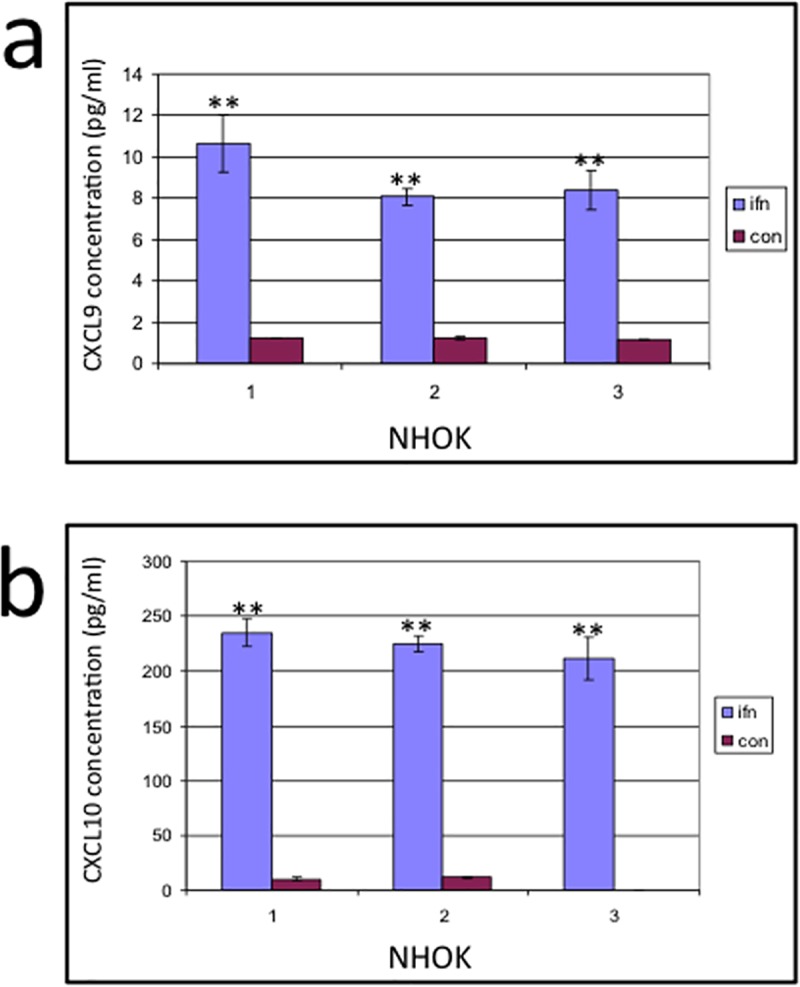
CXCL9/10 production in NHOK strains. In all the NHOK strains CXCL10 production was highly significantly increased after IFN-γ stimulation compared to untreated cells (a). The level of CXCL9 production in the stimulated cells was also highly significantly increased in all the cell lines compared to resting cells, however, there was a substantially lower concentration of CXCL9 produced compared to CXCL10 in all the primary cells (b).

Both CXCL9 and CXCL10 chemokines mRNA were expressed in IFN-γ stimulated primary oral epithelial cells. Interestingly CXCL10, but not CXCL9, displayed low but detectable mRNA expression in the untreated cells (Fig 3B and 3C) (S1 File). These data were confirmed at the protein level by ELISA, which showed a highly significant increase of CXCL10 in all the NHOKs (Fig 4). Weak CXCL11 mRNA expression was witnessed in the treated cells, but was absent in the non-treated cells (Fig 3D).

Taken together, the data show that CXCL9/10/11 are overexpressed in NHOK upon IFN-γ stimulation. In particular, constitutive CXCL10 production was significantly and consistently enhanced by IFN-γ in all three keratinocyte strains.

### CXCL10 induces the migration of specific subsets of T lymphocytes

**Fig 5:**
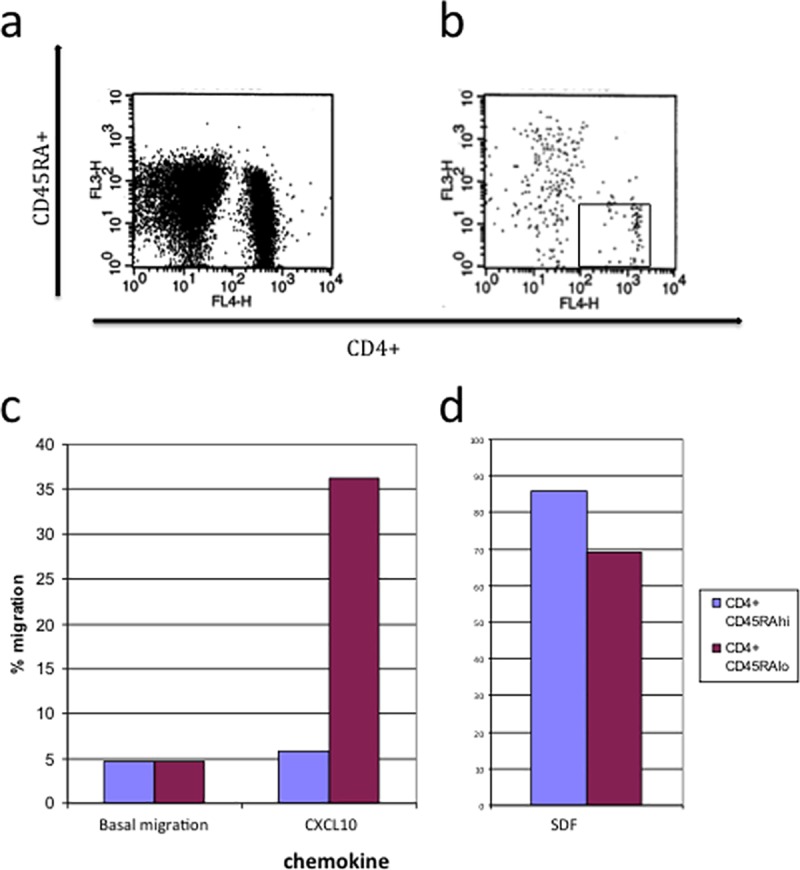
FACS profile of gated lymphocytes labelled with anti-CD4 and anti-CD45RA. migrated to a) 100nM CXCL12 (SDF-1alpha) and b) 1μg/ml CXCL10. c)/d) The normalised % migration of input PBMC to CXCL10 and CXCL12. Basal migration is the migration to cell culture medium alone.

To investigate if CXCL10 could serve as a chemotactic molecule, FACS analysis was used to assess the migration of peripheral blood mononuclear cells (PBMC) in response to this chemokine. The profile of lymphocytes that are attracted to CXCL10 and CXCL12 (SDF-1alpha), another potent CXC chemokine used as control, are shown in Fig 5A and 5B. Whereas there is a number of CD45RA+ and CD45RA- cells attracted to SDF in the CD4+ population (CD4+CD45RA- gated cells are represented by the box on the graphs), CXCL10 cells attract mainly CD4+CD45RA- cells from this population. Both CXCL10 and CXCL12 attract lymphocyte populations above the rate of basal migration, including CD4+CD45RA- cells (Fig 5C and 5D).

### Semi-quantification of CXCL9, CXCL10 and CXCL11 mRNA in OLP and normal oral mucosa

**Fig 6:**
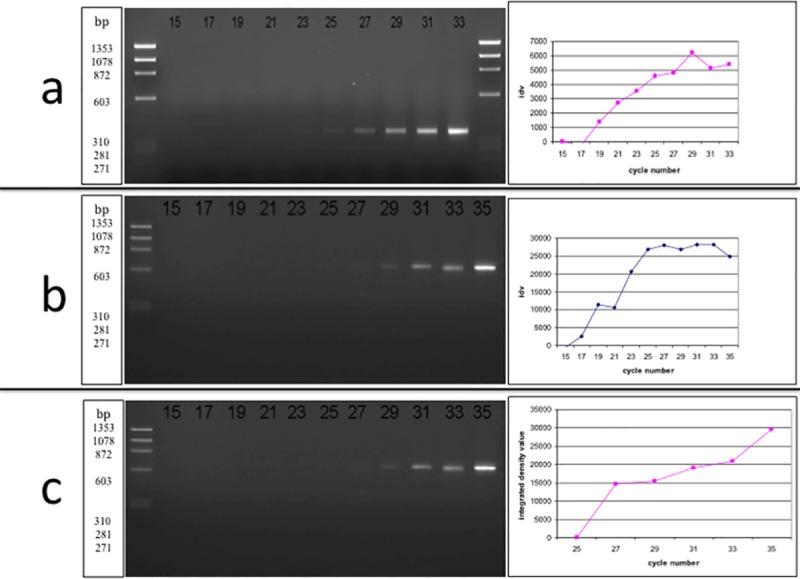
**a) The linear range of CXCL9 (a), CXCL10 (b), CXCL11 (c) mRNA expression in a representative OLP sample.** The samples were removed from the PCR machine every 2 cycles (from cycle 15–33). CXCL9 expression is located at 351 bp. **b)** The same process as for CXCL9 was carried out. The molecular weight of CXCL10 expression is at 601 bp.

Previous studies have failed to demonstrate the expression of CXCL9/10/11 in normal oral mucosa [38, 18, 46], however our results show that primary oral keratinocytes can express some of these chemokines under basal conditions. Therefore, studies to set up optimal experimental conditions were undertaken. The idv (integrated density value) of CXCL9 (Fig 6A), CXCL10 (Fig 6B) and CXCL11 (Fig 6C) mRNA specific bands after removing the RT-PCR reactions at different cycle numbers is shown in Fig 6. The midpoint of the linear ranges of CXCL9, CXCL10 and CXCL11 was calculated to be 23, 21 and 30 respectively, therefore these cycle lengths were utilised for the following semi-quantitative study. The ratio of 18S:competitor mix utilised in all the semi-quantitative analysis was 2:8 respectively as this ratio was found to demonstrate equivalent specific intensities to those without the addition of 18S.

**Fig 7:**
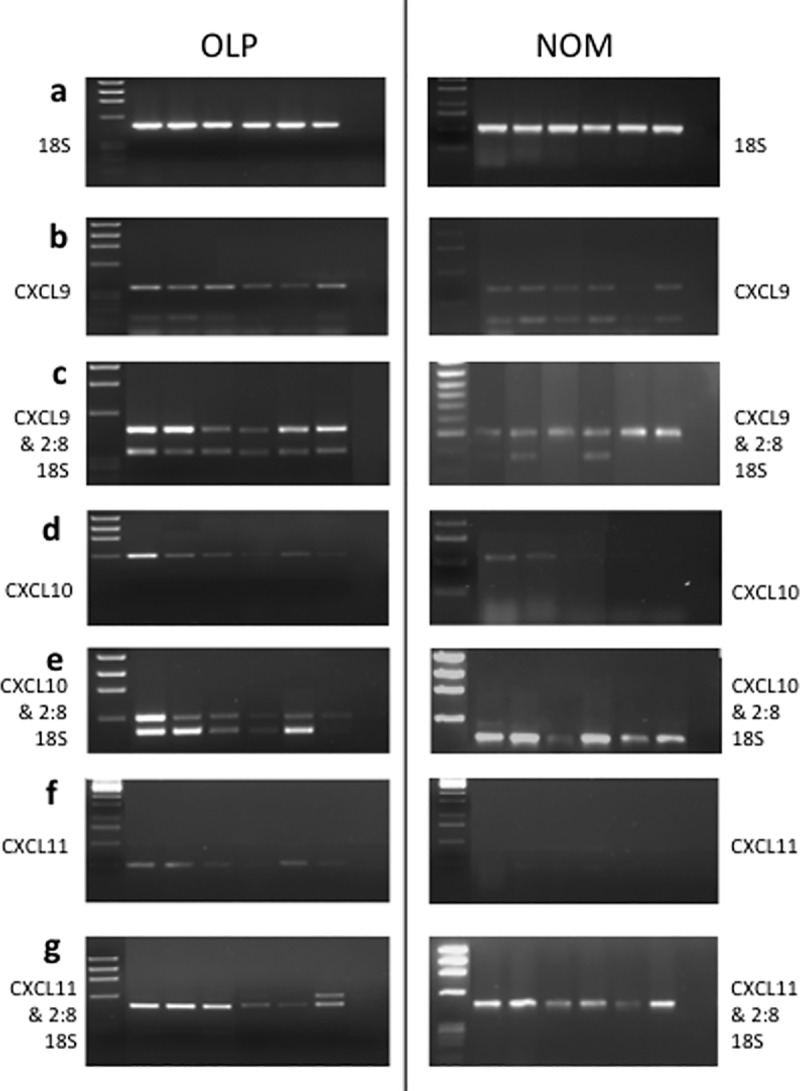
The semi-quantification of the CXC ELR- chemokines in oral inflammation. a) 18S, b) CXCL9, d) CXCL10 and f) CXCL11 mRNA expression in 6 samples of oral lichen planus (OLP) (as a representative of 12 different cases) and 6 samples of normal oral mucosa (NOM). c) CXCL9, e) CXCL10 and g) CXCL11 mRNA expression for the same cases with a 2:8 ratio of 18S:competitor (2:8 18S).

Next, we investigated the expression of CXCL9/10/11 in oral tissues from patients with OLP, an inflammatory disease were IFN-γ is produced at high levels [40]. The greatest ratio of the CXC ELR- chemokines in OLP tissues compared to the adjusted 18S was CXCL9, followed by CXCL10, whereas CXCL11 was only minimally expressed in OLP (Fig 7 and Table 1). However, CXCL11 expression was not detected semi-quantitatively at all in NOM, and all OLP samples expressed CXCL11 without 18S /competitor whereas only faint expression for CXCL11 mRNA could be seen in normal oral mucosa samples. Conversely, CXCL9 and CXCL10 mRNA levels were present in NOM, however their ratios were substantially higher in OLP than NOM tissue.

**Table 1:** The semi-quantitative ratios of MIG (CXCL9), IP-10 (CXCL10) and I-TAC (CXCL11) mRNA expression in OLP and NOM tissue compared to 18S / 2:8 competitor mRNA expression.

				P value
Number of samples quantified	CXCL9	12	6	
	CXCL10	12	6	
	CXCL11	12	6	
Ratio of 2:8 18S:chemokine	CXCL9	0.71 (±0.47)	0.144 (±0.24)	* (p = 0.01066395)
	CXCL10	0.49 (±0.23)	0.166 (±0.27)	* (p = 0.02650378)
	CXCL11	0.048 (±0.121793)	0	p = 0.1808416

± standard deviation of the ratios is shown in brackets. Statistical significance is indicated as * = p<0.05.

Our data demonstrate that normal oral mucosa expresses low levels of CXCL9 and CXCL10 chemokines that are significantly up-regulated in OLP tissues.

## Discussion

This study was the first to demonstrate the induction of CXC ELR- chemokine release in human primary oral keratinocytes as well as oral cancer cells after IFN-γ treatment. Furthermore, the ex-vivo analysis showed higher levels of all three the chemokines CXCL9/10/11 in tissues from patients with OLP compared to normal oral mucosa, reflecting our results from the cell culture model.

We assessed that IFN-γ treatment alone was sufficient to induce both CXCL9 and CXCL10 expression in oral epithelial primary cells. This confirms that oral keratinocytes are a responsive cell type to such selective stimulation. Our results showed that these chemokines are absent or only produced to a minimal degree in resting cells, but are induced after IFN-γ stimulation, suggesting that oral epithelial cells may have more in common in terms of CXC ELR- chemokine production with the skin / bronchial epithelium rather than gut epithelium [9,19,47,48].

IFN-γ-induced CXCL9 and CXCL10 mRNA has previously been found in glomerular cells [20], fibroblasts [15], bronchial epithelial cells [19] and cutaneous epithelial cells [47] whereas CXCL11 mRNA can be induced in primary oral epithelial cells under the influence of IFN-γ, in common with monocytes, astrocytes [49], endothelial cells [17], cutaneous keratinocytes [9,47] and bronchial epithelial cells [19].

In our study the levels of IFN-γ–induced CXCL10 were significantly higher than those of CXCL9 in all primary oral epithelial cells. This was similar in pattern to the findings of Tensen et al., in cutaneous keratinocytes [9]. However, the mRNA appears to decrease in cutaneous keratinocytes after 12hours of stimulation [47], whereas in primary oral keratinocytes and H357 cells there is still mRNA expression at 48hours post-treatment. However, the study of cutaneous keratinocytes stimulated the cells with only 200U/ml IFN-gamma compared to 1000U/ml in this study and this increased concentration may account for the difference

We found that there was a well detectable level of CXCL10 mRNA transcript in unstimulated cells, despite only low level of protein was produced. This correlates with the *in vitro* findings of Boorsma et al. in cutaneous keratinocytes [50], although the level of protein secretion in both the oral and cutaneous cells in this study paralleled with mRNA patterns.

In our study the peak production of both CXCL10 and CXCL9 was at 48 hours after IFN-γ incubation, however, there was an increased production of CXCL10 after only 3 hours of IFN-γ incubation. This pattern correlates with previous studies on stimulated bronchial epithelium supernatant [19] and IFN-γ treated neutrophils [16].

The production of CXCL9/10/11 chemokines in oral cancer cells is also novel.

There is an early induction of CXCL9/10/11 mRNA in H357 cells after just 3 hours of incubation with IFN-γ, and the expression increases until 24 hours for each chemokine. In contrast, there is no or little detectable mRNA for the control, untreated cells.

We found in this study that the prominent IFN-γ-induced chemokine, CXCL10, is chemotactic for lymphocytes and especially T cells with memory cell characteristics. This is in agreement with other studies that found CXCR3 is predominately expressed upon memory CD4 cells (and naïve and memory CD8+ cells) [11,51]. Therefore, production of CXCL10 in the oral epithelium is likely to recruit cells with these characteristics to the epithelial area, and may contribute to inflammatory conditions that are characterised by memory T cell infiltration such as OLP.

A further mainstay of this study is the upregulation of CXC ELR- chemokines mRNA in OLP tissue. CXCL9/10/11 chemokine expression were all up-regulated in OLP compared to normal oral mucosal tissue.

In our study CXCL9 had the highest level of expression compared to 18S in OLP lesional tissue, whereas CXCL11 was weakly expressed compared to CXCL9 and CXCL10. The *ex vivo* results found in this study of OLP are consistent with previous immunohistochemical studies in cutaneous lichen planus [9,31,36], and mucosal lichen planus [31,38,52] where CXCL9 was the dominant chemokine expressed and CXCL11 was only expressed to a small degree. In these studies CXCL9 was mainly found to be expressed by the dermal infiltrate, whereas CXCL10 and CXCL11 was expressed by keratinocytes adjacent to the infiltrate.

In contrast, in other oral inflammatory conditions, such as periodontitis, there appears to be a different chemokine profile with only few biopsy samples demonstrating CXCL10 expression in oral epithelium [39]. However, a study of inflamed gingival tissue found that there were infiltrating CXCR3 cells present [53], suggesting that these cells can play a role in oral inflammation. This differential expression of chemokines in the 2 different oral disorders would suggest there is a recruitment of different cell types, and this is probably important in the pathogenesis of these conditions.

As IFN-γ production is a characteristic feature of Th1 cells, the fact that Th1 type cells are located in this region leads to the possibility that these cells are CXCR3+ cells recruited by the CXC ELR- chemokines. Furthermore, CXCL9/10/11 have shown to be antagonists for CCR3 bearing cells [54], a receptor associated with Th2 cells, which could further skew the reaction in OLP to a Th1 reaction. These recruited Th1 cells could in turn induce the local production of more CXCR3-binding chemokines through IFN-γ leading to the exacerbation of the lesions.

Interestingly, this pattern of chemokine expression in OLP is similar in allergic contact dermatitis [12,40], suggesting that OLP may well be caused by a hypersensitivity reaction to as yet an unknown trigger.

As recent research suggests that OLP may be regarded as an autoimmune condition [52], is it interesting to note that the expression of CXCR3-binding chemokines has also been associated in other T cell mediated autoimmune reactions besides OLP. In a murine model of autoimmune disease the production of CXCL9/10/11 chemokines appears to reduce after 24 days which corresponds to the destruction of the target organs in this condition [32].

CXCL9/10/11 chemokines are also noted to have antimicrobial activity, in a mechanism similar to that of the defensins [55]. As proposed by Thornhill in 2001 [56], an oral autoimmune reaction could possibly be caused by local food antigens or the microbial flora; thus, one can speculate that these chemokines could be induced in the early stages of OLP because of their function as antimicrobial agents, although their chemotactic properties could eventually lead to the large T cell infiltrate witnessed in overt OLP tissues. CXCL9 and CXCL10 are also proposed to have a role in the latter stages of wound healing [57], correlating with a lymphocyte infiltration peaking at 14 days after wound damage.

In conclusion, the finding that there is an up-regulation of CXCL9, CXCL10 and CXCL11 in OLP compared to normal oral mucosa as well as in IFN-γ stimulated oral keratinocytes suggest that these chemokines may play a role in determining the inflammatory infiltrate of OLP, specifically in the migration of specific T cell subsets. CXCL10 was produced in large quantities in oral epithelium in our studies, and was found to attract CD4+CD45RA- T cells, suggesting that this chemokine could have a pivotal role in the accumulation of memory T cells in the band-like infiltrate below the oral epithelium. Further studies are warranted to confirm this novel hypothesis on the pathobiological mechanisms of OLP.

## Supporting information

S1 File (PDF)
