# Supplementary material for: Tissue-specific regulation of CXCL9/10/11 chemokines in keratinocytes: Implications for oral inflammatory disease
Source: PLoS One. 2017 Mar 2;12(3):e0172821. doi: 10.1371/journal.pone.0172821 (PMC5333845; doi:10.1371/journal.pone.0172821)
Supplement: S1 File — (PDF) [file pone.0172821.s001.pdf]

Patients

All OLP tissue was collected from patients that were attending the Oral Medicine Clinic, Eastman Dental Institute. The diagnosis of OLP was assessed clinically by two oral medicine specialists (M.M. and S.P.) according to well established criteria,and confirmed histopathologically. The medical history of the patients included in this study was carefully revised, and didn't include any concomitant pharmacological treatment, hematological anomalies or systemic disease. All normal oral mucosa was obtained from patients attending the Oral Surgery Clinic, Eastman Dental Institute for routine third molar extraction. A total number of 54 samples (36 OLP and 18 NOM) were collected. The internal Ethical Committee of the UCL Eastman Dental Institute approved the study protocol, which was performed in accordance with the tenets of the Declaration of Helsinki. All patients provided written informed consent.

Cell culture techniques

Normal Human Oral keratinocytes (NHOK) cell culture.

Normal oral mucosal tissue was obtained for this study from healthy patients. Three different NHOK strains (NHOK1, NHOK2, NHOK3) were isolated from the excised normal tissue by separating the connective tissue. The samples were cut into approximately 1mm3 pieces and culturing at 37oC /5% CO2 in keratinocyte basal medium-2 containing the recommended growth supplements (Biowittaker, Wokingham, UK). The epithelial cells were then detached using 0.25% trypsin-1mM EDTA. The viability of the keratinocytes was confirmed by trypan blue exclusion.

H357 cell culture

The oral squamous cell carcinoma cell line, H357, was established by Prime et al [41], from a primary explant of a tongue squamous cell carcinoma. This cell line was grown in the same medium as described for the NHOK.

IFN-γ cell treatment assay

In a modification of the method utilised by Altenburg et al [42], the NHOK1, NHOK2, NHOK3 and the H357 cell line (at 2nd or 3rd passage) were seeded at 8x104cells/ well in a Falcon 6 well plate (Becton Dickinson, Oxford, UK) with 3mls of KBM-2 medium containing no hydrocortisone. The cells were incubated for at least 3-5 days until cell culture was 60-80% confluent. We set up the optimal experimental conditions in preliminary experiments with dose-response curves. Medium containing human recombinant 1000U/ml IFN-γ (catalogue number I3265, purity ≥ 98%, Sigma–Aldrich, Poole, UK) was added to 3 wells and control cell culture medium only was added to the remaining 3 wells. The 1000U/ml concentration of IFN-γ had been successfully used by previous studies to stimulate keratinocytes in vitro [42-45]. The cells were incubated for 48hrs or, in the case of the H357 time course, for the following time-points: 3hrs, 6hrs, 9hrs, 24hrs, 48hrs and 72hrs. The supernatant was extracted, centrifuged and stored at –70oC. The adherent cells were washed with PBS (Gibco Life Technologies, Paisley, UK) before 0.5ml of Trireagent were added. The suspension was then removed and stored at –70oC. The RNA was isolated as described below.

mRNA isolation and semi-quantitative RT-PCR

OLP and normal oral mucosa (NOM) tissue were obtained and prepared for RNA isolation. RNA isolation and cDNA synthesis of NHOK, H357, NOM and OLP tissue

was carried out. The RNA was extracted according to the manufacturer's instructions, utilising 2ul Pellet Paint Co-precipitant (Novagen, Nottingham, UK) to visualise the RNA pellet. The purified RNA was dissolved into 25ul DEPC water (Ambion, Austin, US) and stored at -70°C.

All procedures for the cDNA Single strand synthesis were carried out on ice. 2ul of RNA was added to 4ul deoxynucleotides (dNTPs) (2.5mM) (Sigma), 2ul of random hexamers (50um) (Ambion, Texas, USA) and 9.5ul dH2O. This was incubated at 70°C for 3 minutes and allowed to cool at room temperature. Then 1ul of RNAaseIN (Ambion, Austin, Texas, USA), 2ul 10x MuLVRT buffer and 0.5ul M-MuLVRT (200U/ul) (Boehringer-Mannheim, Germany) was added and incubated at 42°C for 1 hour. cDNA samples were stored at –20°C.

CXCL9 (5'-ccaacaccccacagaagtgc–3', 5'-gccagcacctgctctgagac-3'),

CXCL10 (5'- gccaattttgtccacgtgttg-3', 5'-aaagaatttgggccccttgg-3'),

CXCL11 (5'-ggcttccccatgttcaaag –3', 5'-cagatgcccttttccaggac-3') and

primers were generated for use in this study (Genosys-Sigma, Poole, UK). The thermocycler (Techne Genius, Cambridge, UK) parameters utilised were at 94oC for 45secs, 57oC for 45secs, 72oC for 45secs.

CXCL9/10/11 primers (as described above) were utilised with QuantamRNA 18S internal standards (Ambion, Texas, USA). The primers utilised for the study of housekeeping expression encoded a region of 18S ribosomal RNA (5'-tttcggaactgaggccatga-3', 5'- gcatgccagagtctcgttcg -3').

For each primer the linear range was determined by repeating the above reaction with optimised magnesium concentration for each primer and stopping the reaction at 15, 17, 19, 21, 23, 25, 27, 29, 31, 33 and 35 cycles. The mid-point of each linear range was determined by using intensity analysis of the bands with AlphaImager software,

and this cycle length was utilised for each primer in subsequent reactions. 18S primer and 18S Competitor primers (Ambion, Texas, USA) were combined to ratios 1:9, 2:8 and 3:7 respectively. For each of the primers (CXCL9/10/11) 4µl of the 18S primer competitor mix was added to the RT-PCR reaction and the results were compared to the reactions containing the specific primers without any 18S primer. The reaction that had the same level of specific primer expression as that without 18S primer added was selected for quantification. The band intensity of the 18S and of the specific primer was quantified in each sample with Phoretix 1D software (Phoretix, Newcastle, UK).

Enzyme-linked immunosorbant assay (ELISA)

A 96 well maxisorp-surface immunoplate (Nunc, Denmark) was coated overnight with a monoclonal antibody against the human protein to be studied. The plate was then washed 3 times with wash buffer (2.5mMNa_2HPO_4 (BDH), 0.5mM NaCl (BDH), 7.5mM $NaH_2PO_4.2H_2O$ (BDH) and 0.1% of Tween 20 (BDH). 100ml of cell supernatant or positive control (in a range of dilutions to obtain a standard curve) was added and incubated for 2 hours at room temperature then washed. A biotinylated antibody was used as a secondary antibody; 100µl of this antibody, diluted to an appropriate concentration, was added to each well. The plate was sealed and incubated for 1 hour at room temperature, then washed 3 times. Bound secondary antibody was detected by adding 100µl avidin-HRP (Dako, Denmark) [diluted 1:4000] and incubating for 30 minutes at room temperature. 25ml H_2O_2 was added to OPD (1 tablet of o-phenyl diaminazadine (Sigma, Poole, Dorset) in 25ml of 34.7mM citric acid, 66.7mM Na_2HPO_4) and 100µl of this solution was dispensed to each well immediately and incubated at room temperature for 15 mins. The reaction

was stopped by adding 100ml of 1M sulphuric acid to the wells and the absorbance measured at 490nM. Chemokine concentration in the supernatant was then extrapolated from the standard curve generated from standards using Revelation software (Dynex Technologies, Virginia, US) attached to an ELISA plate reader (Dynex Technologies, Virginia, US).

Chemotaxis assay

Peripheral blood mononuclear cells (PBMC) were prepared from fresh blood obtained from healthy patients. The lymphocyte separation was carried using Ficoll-Paque (Amersham) according to the manufacturers instructions. Peripheral blood lymphocytes obtained were incubated for 1hr in RPMI-1640 (Gibco Life Technologies, Paisley, UK) plus 5% foetal bovine serum (Sigma, Poole, UK), and those cells remaining in suspension were adjusted to a density of 5x106 cells/ml. The cells were then migrated towards 1µg/ml recombinant human CXCL10 or 100mM SDF-1alpha (R&D Systems, Minneapolis, US) in a transwell migration assay. 600µl of cell culture supernatant from each sample was added to the bottom chamber of Corning Co-star 5µm pore transwells (BDH, UK) in triplicate. 100µl of PBL was added to the top chamber. The transwells were then incubated for 3 hours at 37oC in atmosphere containing 5% CO2. After migration, the cells that passed through the membrane were collected and incubated with allophycocyanin conjugated anti-human CD45RA (Clone HI100) and Cy-chrome conjugated mouse anti-human CD4 (Clone RPA-T4) (Both BDPharmingen, San Diego, US) for 30 mins at 3µl /106 cells. The migrated cell populations were then analysed using a florescence activated cell sorter (FACS) machine (Becton Dickinson, Oxford, UK).

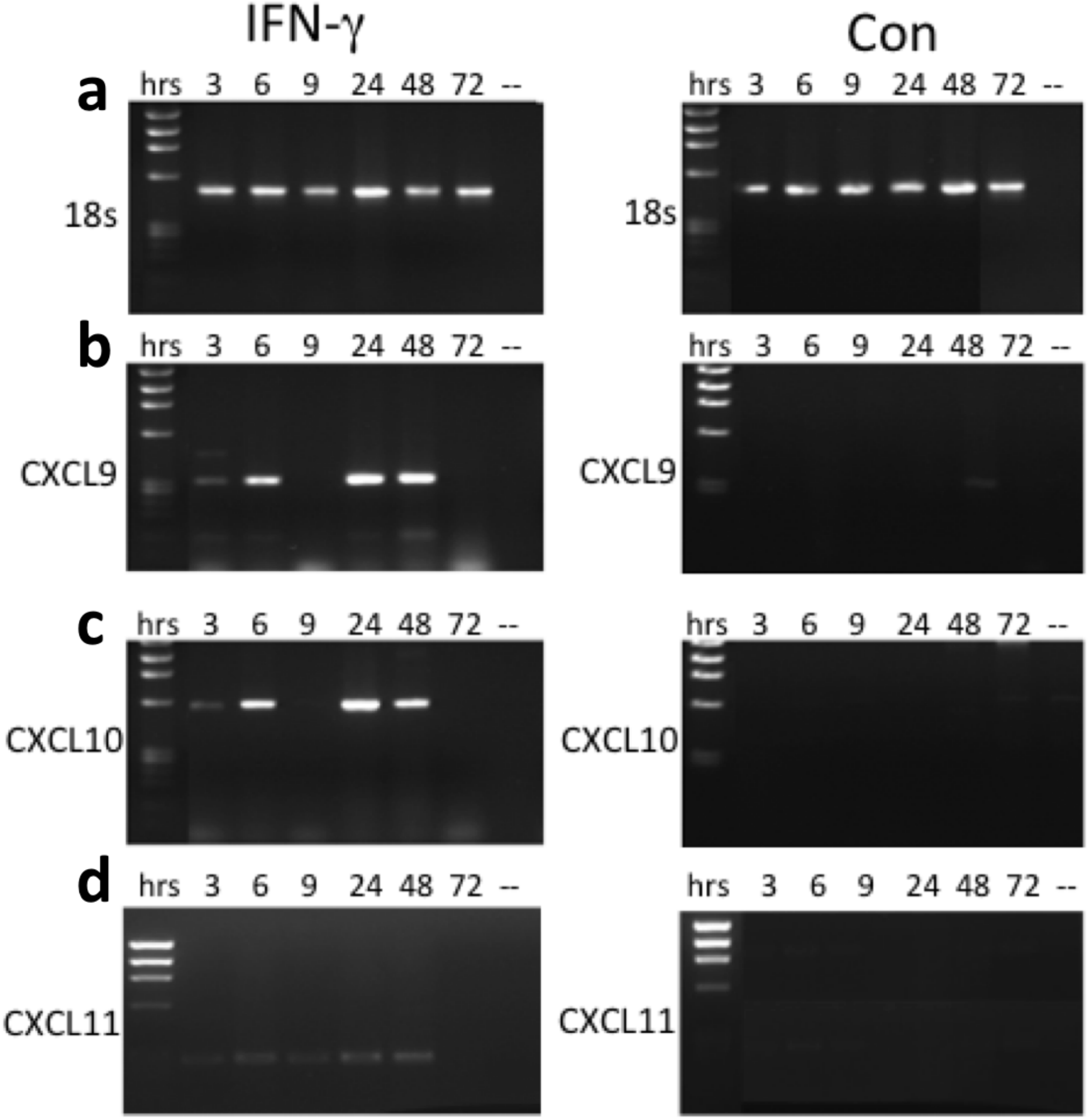
IFN-γ
Con
a
b
c
d
hrs 3 6 9 24 48 72 --
18s
CXCL9
CXCL10
CXCL11

a

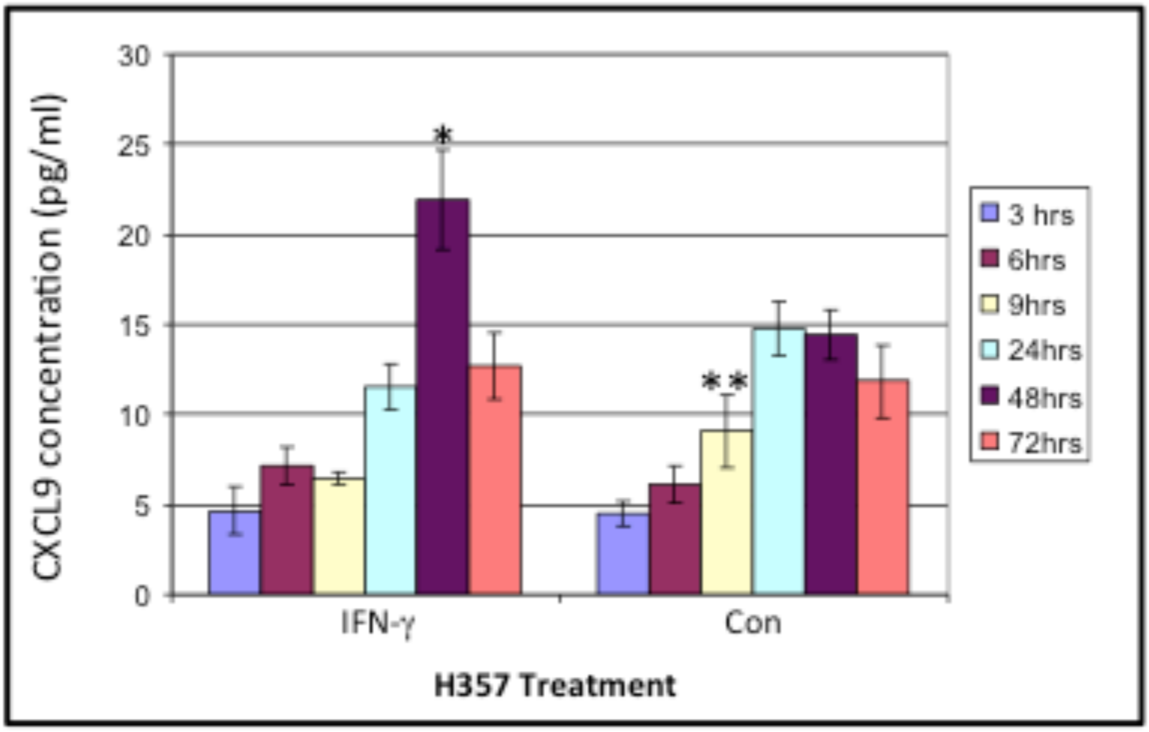

CXCL9 concentration (pg/ml)
30
25
20
15
10
5
0
IFN-γ
Con
H357 Treatment
3 hrs
6hrs
9hrs
24hrs
48hrs
72hrs

b

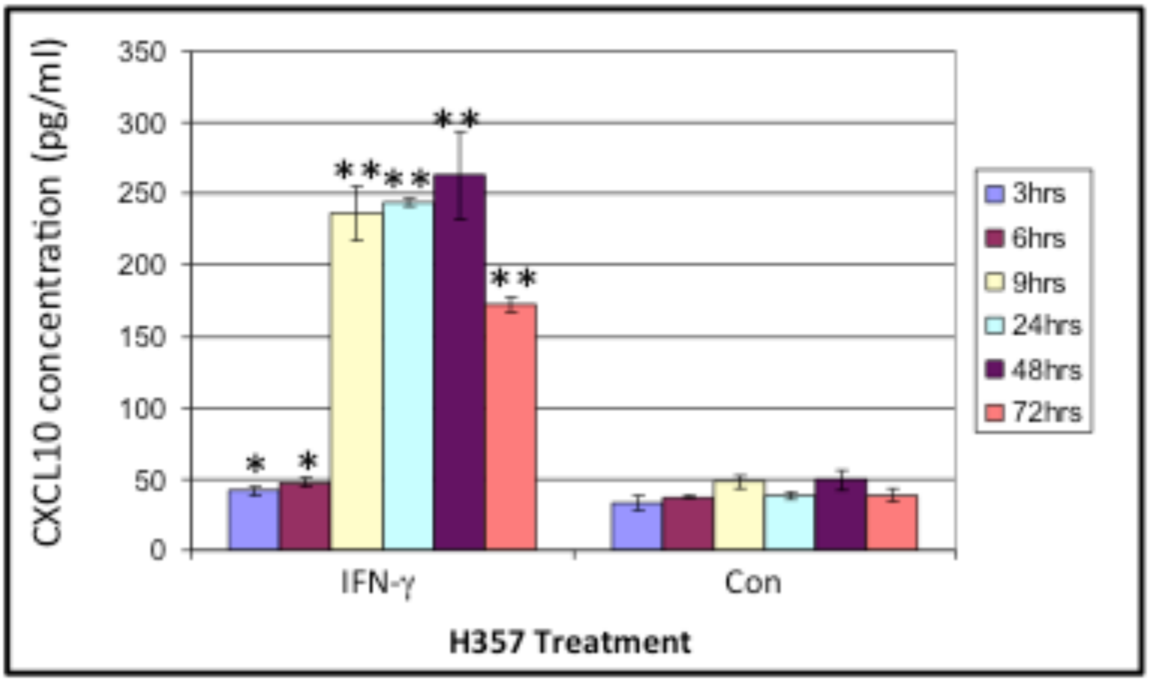

CXCL10 concentration (pg/ml)
350
300
250
200
150
100
50
0
IFN-γ
Con
H357 Treatment
3hrs
6hrs
9hrs
24hrs
48hrs
72hrs

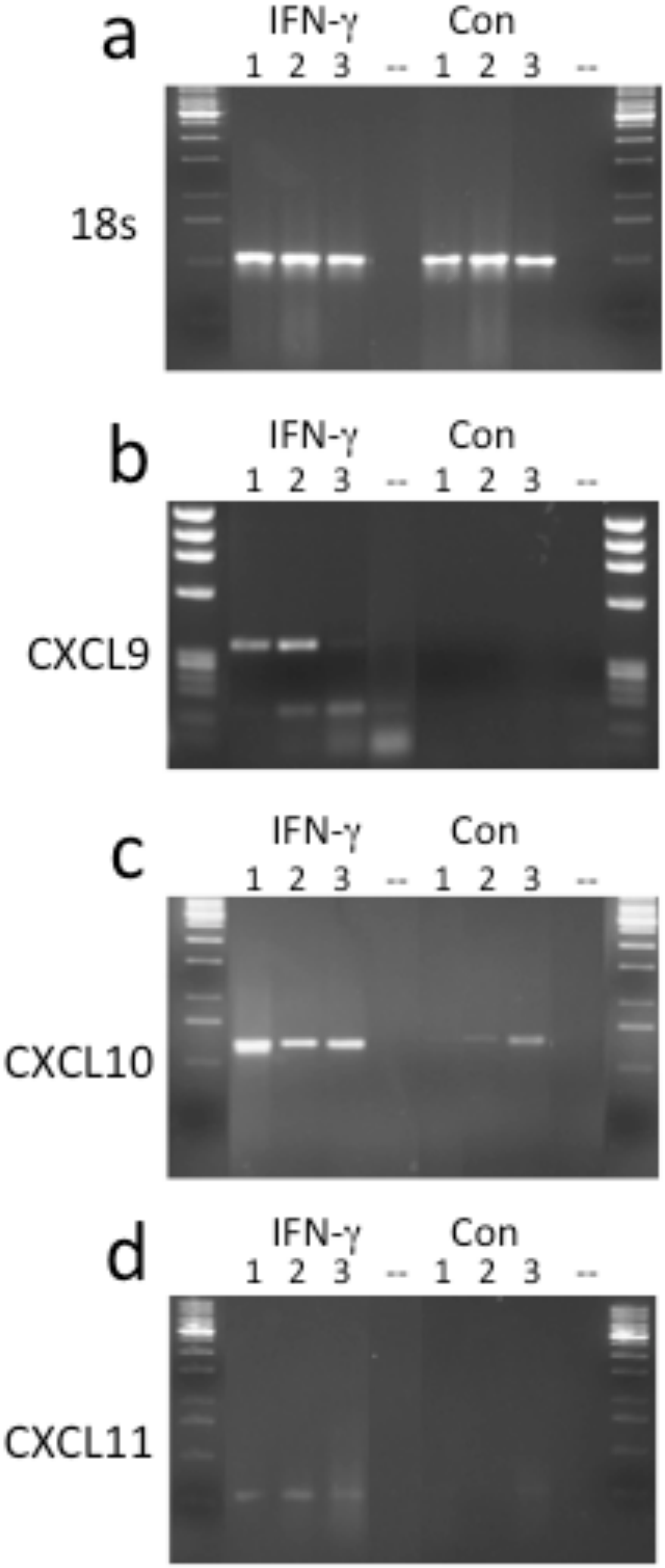

a
IFN-γ
Con
1 2 3 -- 1 2 3 --
18s
b
IFN-γ
Con
1 2 3 -- 1 2 3 --
CXCL9
c
IFN-γ
Con
1 2 3 -- 1 2 3 --
CXCL10
d
IFN-γ
Con
1 2 3 -- 1 2 3 --
CXCL11

a

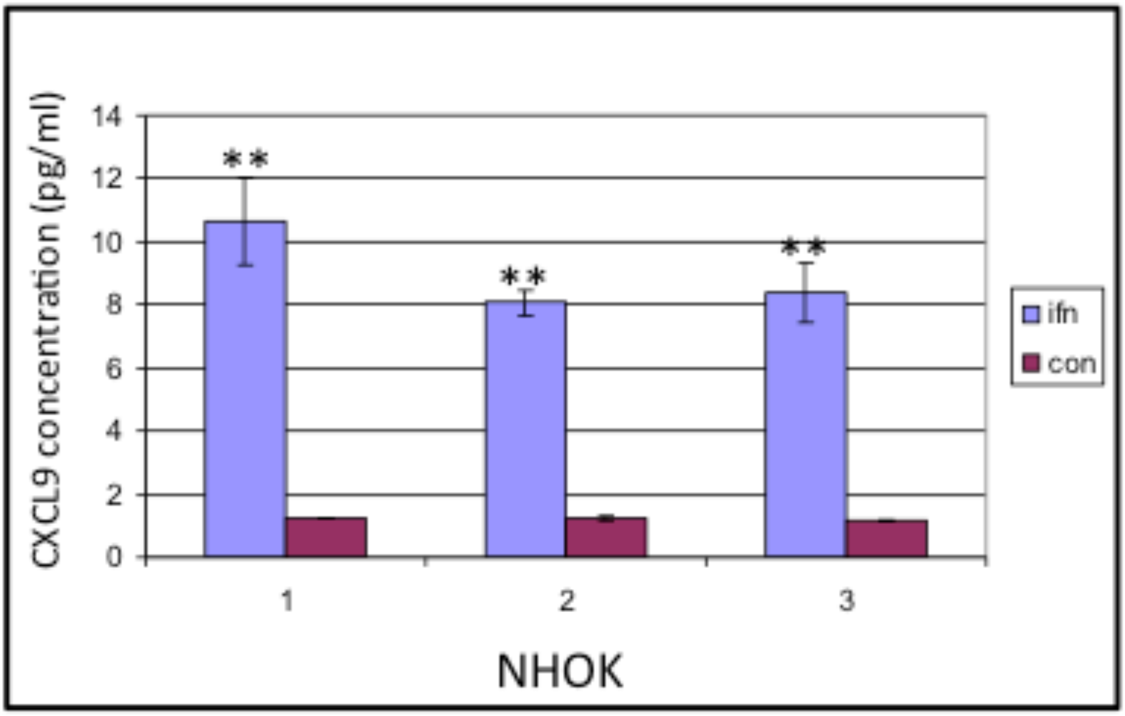

CXCL9 concentration (pg/ml)
14
12
10
8
6
4
2
0
**
**
**
1
2
3
NHOK
ifn
con

b

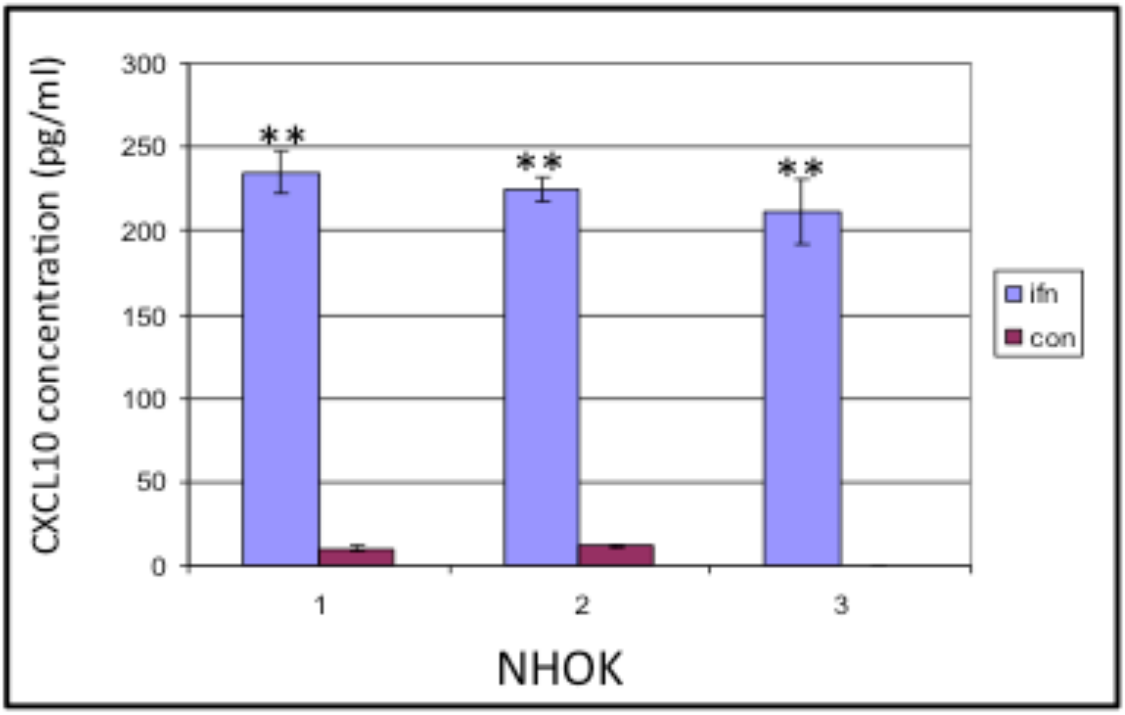

CXCL10 concentration (pg/ml)
300
250
200
150
100
50
0
**
**
**
1
2
3
NHOK
ifn
con

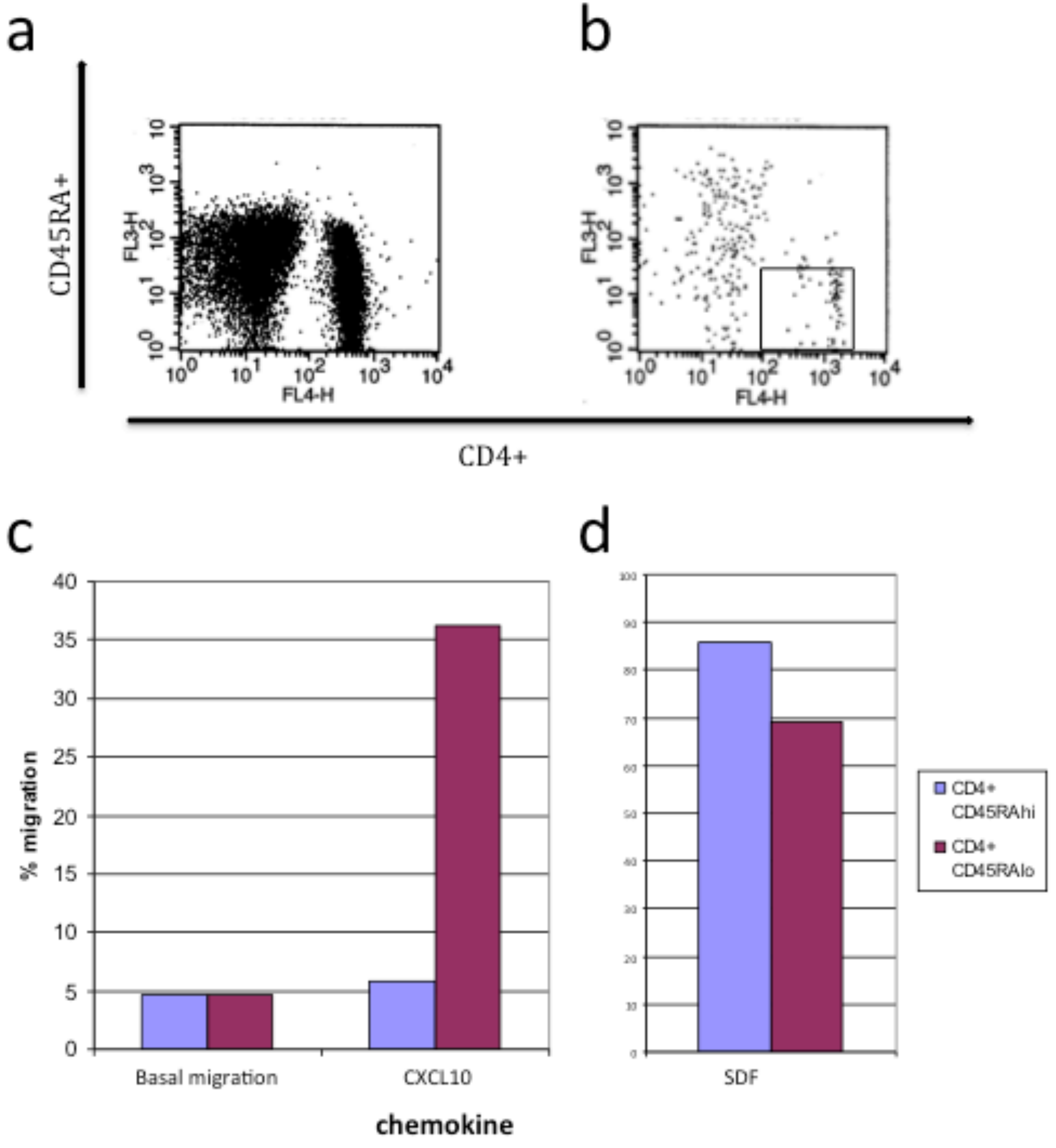
a
b
CD45RA+
FL3-H
FL4-H
CD4+
c
d
% migration
Basal migration
CXCL10
chemokine
SDF
CD4+ CD45RAhi
CD4+ CD45RAlo

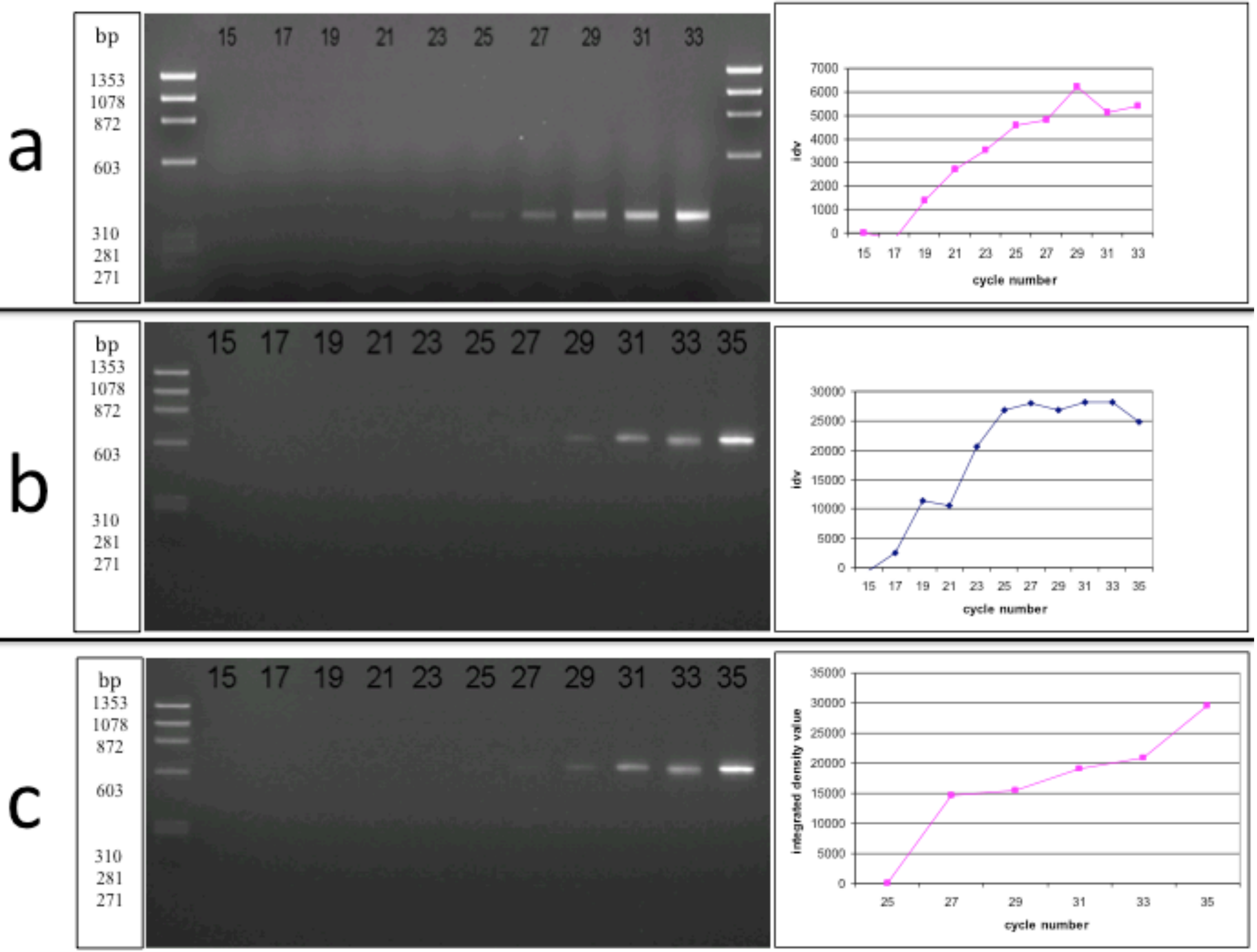
a
b
c
bp
1353
1078
872
603
310
281
271
15 17 19 21 23 25 27 29 31 33 35
idv
integrated density value
cycle number
7000
6000
5000
4000
3000
2000
1000
0
30000
25000
20000
15000
10000
5000
35000

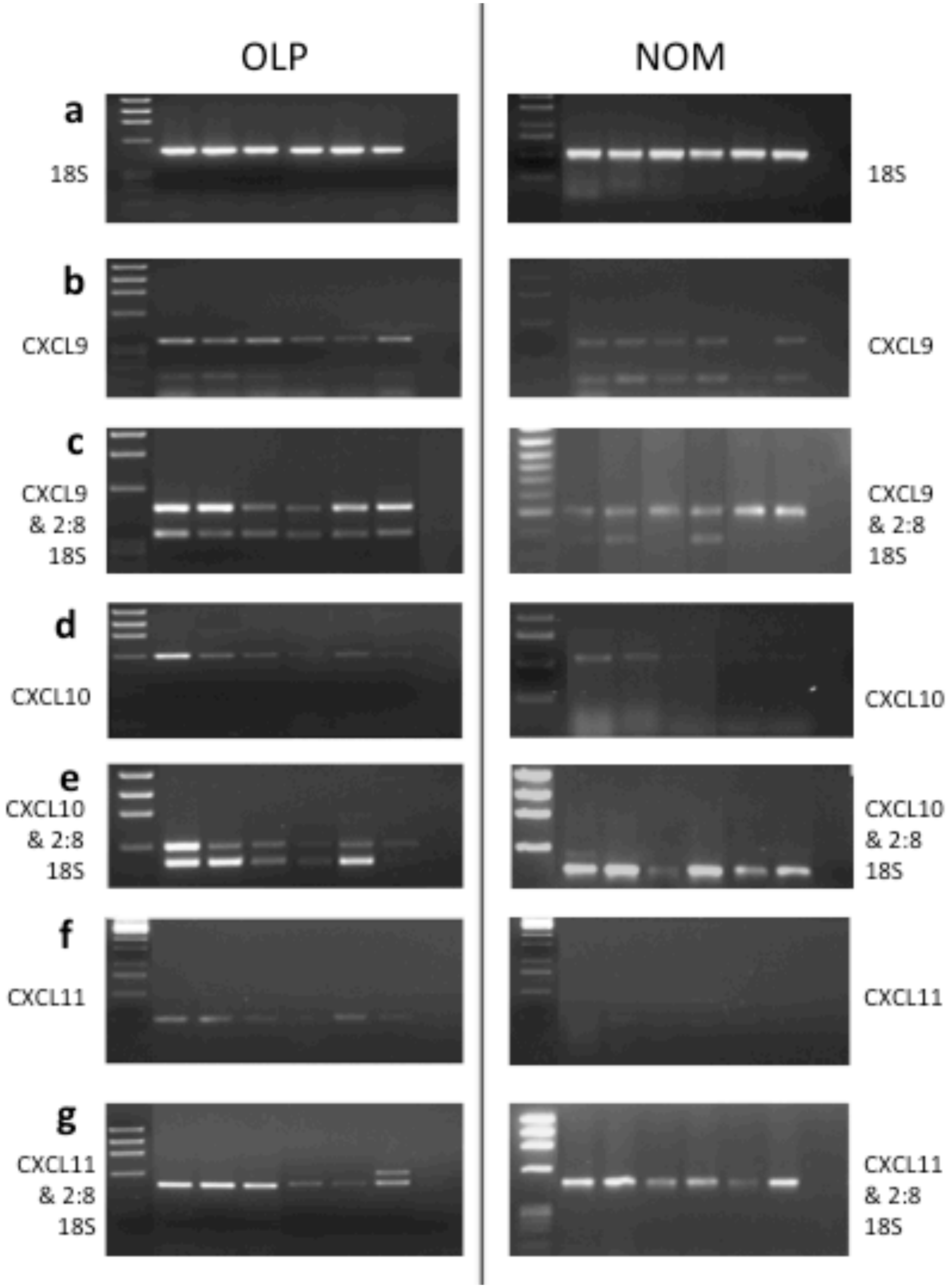
OLP
NOM
a
18S
18S
b
CXCL9
CXCL9
c
CXCL9
& 2:8
18S
CXCL9
& 2:8
18S
d
CXCL10
CXCL10
e
CXCL10
& 2:8
18S
CXCL10
& 2:8
18S
f
CXCL11
CXCL11
g
CXCL11
& 2:8
18S
CXCL11
& 2:8
18S

		OLP	NOM	p value
**Number of samples quantified**	CXCL9	12	6	
	CXCL10	12	6	
	CXCL11	12	6	
**Ratio of 2:8 18S: chemokine**	CXCL9	0.71 (± 0.47)	0.144 (± 0.24)	* (p= 0.01066395)
	CXCL10	0.49 (± 0.23)	0.166 (± 0.27)	* (p= 0.02650378)
	CXCL11	0.048 (± 0.121793)	0	p= 0.1808416

Table 1: The semi-quantitative ratios of MIG (CXCL9), IP-10 (CXCL10) and I-TAC (CXCL11) mRNA expression in OLP and NOM tissue compared to 18S / 2:8 competitor mRNA expression. ±standard deviation of the ratios is shown in brackets.

			18s	cxcl9	cxcl10
24161.054	18727.054	26425.598	1		
25160.811	22429.933	16664.861	1		
21837.983	3529.012	17411.79	1		
20657.811	1805.648	2670.113	1		
24587.347	2075.77	3194.113	1		
19774.933	4069.477	6763.205	1		

Con	0.08740752	0.08442432	0.20578967
IFN-γ	0.77509259	0.89146304	0.16159972
Con	0.1292544	0.12990881	0.342009
IFN-γ	1.09372704	0.66233402	0.79731677

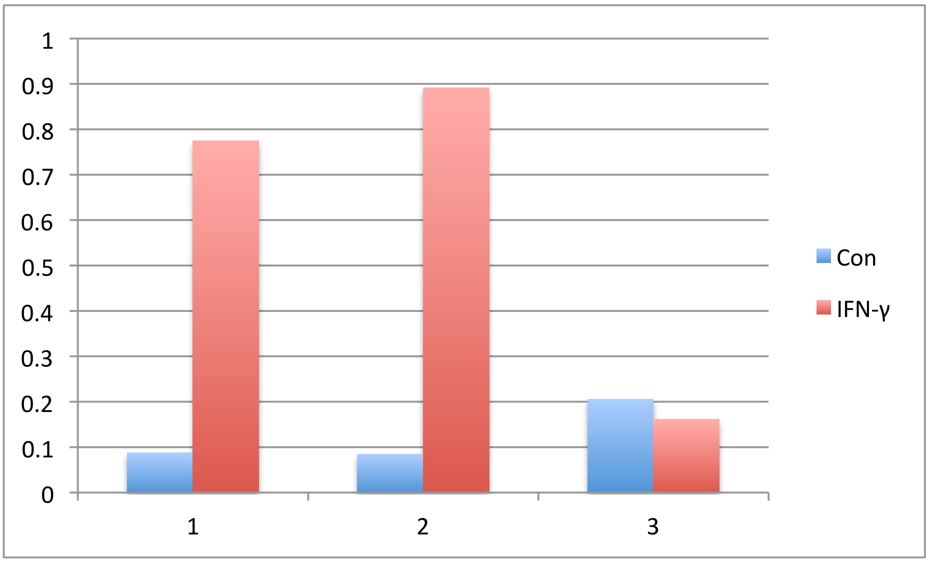

1
0.9
0.8
0.7
0.6
0.5
0.4
0.3
0.2
0.1
0
1
2
3
Con
IFN-γ

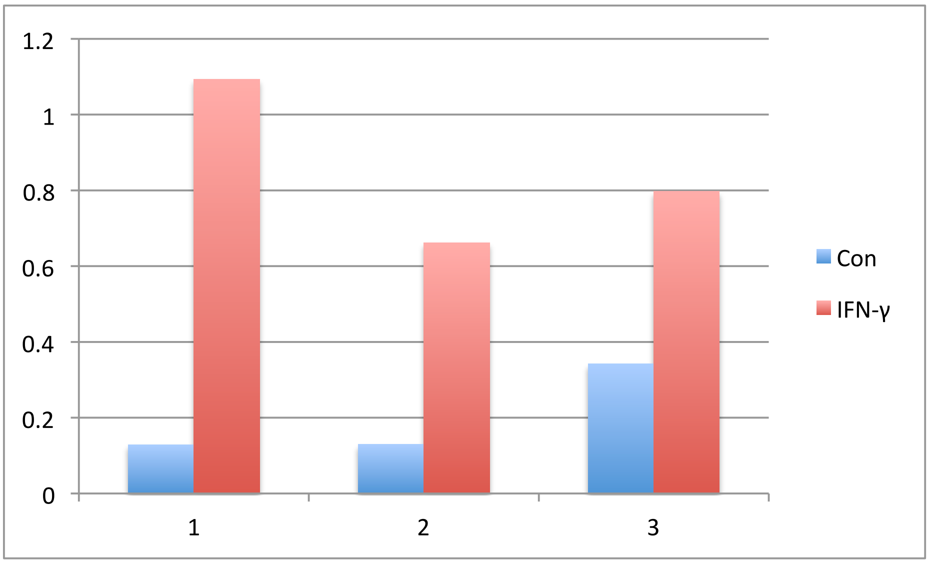

1.2
1
0.8
0.6
0.4
0.2
0
1
2
3
Con
IFN-γ
